# Supplementary material for: Synergy between RecBCD subunits is essential for efficient DNA unwinding
Source: eLife. 2019 Jan 2;8:e40836. doi: 10.7554/eLife.40836 (PMC6338465; doi:10.7554/eLife.40836)
Supplement: Supplementary file 1. [file elife-40836-supp1.docx]

**Supplementary Table 1:** All the possible mechano-chemical models.

| Model | **Reaction scheme** |
| --- | --- |
| PS at Binding | $R_{n}+T\begin{matrix} k_{+b} \\ \rightleftharpoons\\ k_{-b} \end{matrix}R_{n+1}\cdot T\begin{matrix} k_{h} \\ \to\\ \end{matrix}R_{n+1}\cdot D\cdot Pi\begin{matrix} k_{r} \\ \to\\ \end{matrix}R_{n+1}+D+Pi$ |
| PS at hydrolysis | $R_{n}+T\begin{matrix} k_{+b} \\ \rightleftharpoons\\ k_{-b} \end{matrix}R_{n}\cdot T\begin{matrix} k_{h} \\ \to\\ \end{matrix}R_{n+1}\cdot D\cdot Pi\begin{matrix} k_{r} \\ \to\\ \end{matrix}R_{n+1}+D+Pi$ |
| PS at release | $R_{n}+T\begin{matrix} k_{+b} \\ \rightleftharpoons\\ k_{-b} \end{matrix}R_{n}\cdot T\begin{matrix} k_{h} \\ \to\\ \end{matrix}R_{n}\cdot D\cdot Pi\begin{matrix} k_{r} \\ \to\\ \end{matrix}R_{n+1}+D+Pi$ |
| BR before binding | $R_{n}\begin{matrix} k_{+tr} \\ \rightleftharpoons\\ k_{-tr} \end{matrix}R_{n+1}+T\begin{matrix} k_{+b} \\ \rightleftharpoons\\ k_{-b} \end{matrix}R_{n+1}\cdot T\begin{matrix} k_{h} \\ \to\\ \end{matrix}R_{n+1}\cdot D\cdot Pi\begin{matrix} k_{r} \\ \to\\ \end{matrix}R_{n+1}+D+Pi$ |
| BR before hydrolysis | $R_{n}+T\begin{matrix} k_{+b} \\ \rightleftharpoons\\ k_{-b} \end{matrix}R_{n}\cdot T\begin{matrix} k_{+tr} \\ \rightleftharpoons\\ k_{-tr} \end{matrix}R_{n+1}\cdot T\begin{matrix} k_{h} \\ \to\\ \end{matrix}R_{n+1}\cdot D\cdot Pi\begin{matrix} k_{r} \\ \to\\ \end{matrix}R_{n+1}+D+Pi$ |
| BR before release | $R_{n}+T\begin{matrix} k_{+b} \\ \rightleftharpoons\\ k_{-b} \end{matrix}R_{n}\cdot T\begin{matrix} k_{h} \\ \to\\ \end{matrix}R_{n}\cdot D\cdot Pi\begin{matrix} k_{+tr} \\ \rightleftharpoons\\ k_{-tr} \end{matrix}R_{n+1}\cdot D\cdot Pi\begin{matrix} k_{r} \\ \to\\ \end{matrix}R_{n+1}+D+Pi$ |
