# Supplementary material for: Synergy between RecBCD subunits is essential for efficient DNA unwinding
Source: eLife. 2019 Jan 2;8:e40836. doi: 10.7554/eLife.40836 (PMC6338465; doi:10.7554/eLife.40836)
Supplement: Supplementary file 2. [file elife-40836-supp2.docx]

**Supplementary Table 2:** Force-dependent Michaelis Menten parameters for the different models of unwinding, for experiments measuring activity against an opposing force.

| **Model** | $\boldsymbol{v}_{\boldsymbol{max}}$ | **Eq.** | $\frac{\boldsymbol{v}_{\boldsymbol{max}}}{\boldsymbol{K}_{\boldsymbol{M}}}$ | **Eq.** |
| --- | --- | --- | --- | --- |
| PS at Binding | $\frac{k_{r}k_{h}}{k_{r}+k_{h}}$ | (S1) | $\frac{k_{b,0}k_{h}}{k_{-b,0}\exp\left( \frac{F\delta}{k_{b}T} \right)+k_{h}\exp\left( \frac{Fx^{\ddagger}}{k_{b}T} \right)}$ | (S2) |
| PS at hydrolysis | $\frac{1}{\frac{1}{k_{r}}+\frac{1}{k_{h,0}}\exp\left( \frac{Fx^{\ddagger}}{k_{b}T} \right)}$ | (S3) | $\frac{k_{b}}{1+\frac{k_{-b}}{k_{h,0}}\exp\left( \frac{Fx^{\ddagger}}{k_{b}T} \right)}$ | (S4) |
| PS at release | $\frac{k_{h}}{1+\frac{k_{h}}{k_{r,0}}\exp\left( \frac{Fx^{\ddagger}}{k_{b}T} \right)}$ | (S5) | $\frac{k_{b}k_{h}}{k_{-b}+k_{h}}$ | (S6) |
| BR before binding | $\frac{k_{c}^{eff}}{1+\frac{k_{c}^{eff}}{k_{tr,0}}\exp\left( \frac{Fx^{\ddagger}}{k_{b}T} \right)}$ | (S7) | $\frac{\frac{k_{b}k_{c}^{eff}}{(k_{c}^{eff} + k_{-b})}}{1+\frac{k_{-tr}}{k_{tr,0}}\exp\left( \frac{F\delta}{k_{b}T} \right)}$ | (S8) |
| BR before hydrolysis | $\frac{k_{c}^{eff}}{1+\frac{k_{-tr}}{k_{tr,0}}\exp\left( \frac{F\delta}{k_{b}T} \right)+\frac{k_{c}^{eff}}{k_{tr,0}}\exp\left( \frac{Fx^{\ddagger}}{k_{b}T} \right)}$ | (S9) | $\frac{k_{b}}{1+\frac{k_{-b}}{k_{c}^{eff}}\frac{k_{-tr}}{k_{tr,0}}\exp\left( \frac{F\delta}{k_{b}T} \right)+\frac{k_{-b}}{k_{tr,0}}\exp\left( \frac{Fx^{\ddagger}}{k_{b}T} \right)}$ | (S10) |
| BR before release | $\frac{k_{c}^{eff}}{1+k_{c}^{eff}\left[ \frac{1}{k_{r}}\frac{k_{-tr}}{k_{tr,0}}\exp\left( \frac{F\delta}{k_{b}T} \right)+\frac{1}{k_{tr,0}}\exp\left( \frac{Fx^{\ddagger}}{k_{b}T} \right) \right]}$ | (S11) | $\frac{k_{b}k_{h}}{k{}_{h} + k_{-b}}$ | (S12) |
