# Supplementary material for: Synergy between RecBCD subunits is essential for efficient DNA unwinding
Source: eLife. 2019 Jan 2;8:e40836. doi: 10.7554/eLife.40836 (PMC6338465; doi:10.7554/eLife.40836)
Supplement: Supplementary file 3. [file elife-40836-supp3.docx]

**Supplementary Table 3:** Force-dependent Michaelis Menten parameters for the different models of unwinding, for experiments measuring unwinding of a hairpin under tension.

| **Model** | $\boldsymbol{v}_{\boldsymbol{max}}$ | **Eq.** | $\frac{\boldsymbol{v}_{\boldsymbol{max}}}{\boldsymbol{K}_{\boldsymbol{M}}}$ | **Eq.** |
| --- | --- | --- | --- | --- |
| PS at Binding | $\frac{k_{r}k_{h}}{k_{r}+k_{h}}$ | (S13) | $\frac{k_{b,o}k_{h}}{k_{-b}+k_{h}}\cdot P_{open}$ | (S14) |
| PS at hydrolysis | $\frac{k_{r}k_{h,0}}{k_{h,0}+\frac{k_{r}}{P_{open}}}$ | (S15) | $\frac{k_{b}k_{h,0}}{k_{h,0}+\frac{k_{-b}}{P_{open}}}$ | (S16) |
| PS at release | $\frac{k_{2}k_{r,0}}{k_{r,0}+\frac{k_{h}}{P_{open}}}$ | (S17) | $\frac{k_{b}k_{h}}{k_{-b}+k_{h}}$ | (S18) |
| BR before binding | $\frac{k_{c}^{eff}k_{tr,0}}{k_{tr,0}+\frac{k_{c}^{eff}}{P_{open}}}$ | (S19) | $\frac{k_{b}k_{tr,0}k_{c}^{eff}}{k_{tr,0}\left( k_{c}^{eff}+k_{-tr} \right)+\frac{k_{-b}\left( k_{c}^{eff}+k_{-tr} \right)}{P_{open}}}$ | (S20) |
| BR before hydrolysis | $\frac{k_{tr,0}k_{c}^{eff}}{k_{tr,0}+\frac{k_{c}^{eff}+k_{-tr}}{P_{open}}}$ | (S21) | $\frac{k_{b}k_{tr,0}k_{c}^{eff}}{k_{tr,0}k_{c}^{eff}+\frac{k_{-b}\left( k_{c}^{eff}+ k_{-tr} \right)}{P_{open}}}$ | (S22) |
| BR before release | $\frac{1}{\frac{1}{k_{h}}+\frac{1}{k_{r}}+\frac{k_{r} +k_{-tr}}{k_{r}k_{tr,0}P_{open}}}$ | (S23) | $\frac{k_{b}k_{h}}{k{}_{h} + k_{-b}}$ | (S24) |
