# Supplementary material for: Synergy between RecBCD subunits is essential for efficient DNA unwinding
Source: eLife. 2019 Jan 2;8:e40836. doi: 10.7554/eLife.40836 (PMC6338465; doi:10.7554/eLife.40836)
Supplement: Supplementary file 4. [file elife-40836-supp4.docx]

**Supplementary Table 4:** Number of traces measured

|  |  | **ATP (μM)** | | | | | | | | |
| --- | --- | --- | --- | --- | --- | --- | --- | --- | --- | --- |
|  |  | **20** | **50** | **75** | **100** | **200** | **350** | **500** | **1000** | **2000** |
| **Figure 2a** | **RecBCD** | 10 |  |  | 30 | 8 | 25 | 10 | 29 | 34 |
| **Figure 2b** | **RecD** | 5 |  |  | 16 | 18 | 28 | 48 | 24 | 52 |
| **Figure 2c** | **RecB** | 18 |  |  | 13 | 30 | 26 | 30 | 29 | 52 |
| **Figure 2-figure supplement 1a** | **RecB^K29Q^CD** | - |  |  | - | - | - | - | - | 19 |
| **Figure 2-figure supplement 1b** | **RecBC** | - |  |  | - | - | - | - | - | 9 |
| **Figure 3c** | **RecD** |  | 43 | 37 |  | 35 |  |  |  | 77 |
