# Supplementary material for: Synergy between RecBCD subunits is essential for efficient DNA unwinding
Source: eLife. 2019 Jan 2;8:e40836. doi: 10.7554/eLife.40836 (PMC6338465; doi:10.7554/eLife.40836)
Supplement: Supplementary file 5. [file elife-40836-supp5.docx]

**Supplementary Table 5:** Oligonucleotides used for synthesizing DNA substrates for optical tweezers experiments.

| **Construct Name** | **Oligonucleotide sequence (5’→3’)** |
| --- | --- |
| Biotin 600 bp track | /5’Biotin/-GCTTTAATGCGGTAGTTTATCA |
|  | GCAGCATTAGGAAGCAGCCCAGGCATTAGGAAGCAGCCCAG |
| Dig 600 bp track | /5’Phosphate/-GCATTAGGAAGCAGCCCAGGCTTTATTGCGGTAG TTTATCA |
|  | /5’digoxigenin/-GCATTAGGAAGCAGCCCAG |
| Biotin 4000 bp track | /5’Biotin/-GCTTTAATGCGGTAGTTTATCA |
|  | GCACTACGCCTCAGCTTGCCCCTCAGCGATGACCTCAGCATTCCCTTTTTTGCGGCATT |
| Dig 4000 bp track | /5’Phosphate/-CTACGCCTCAGCTTGCCCCTCAGCGATGACCTCAGCGTCACTGGTCCCG |
|  | /5’digoxigenin/-GCATTAGGAAGCAGCCCAG |
| Biotin short track | /5’Biotin/AACCACCAACCAACAACCACCCAAACCCAAACCCAAGGTCATCGCTGAGGGGC AAGCTGAGGCGTAGTGC |
| Dig short track | /5’Phasphate/CTACGCCTCAGCTTGCCCCTCAGCGATGACCAAACCACCAACCAACAACCACCCAAACCCAAACCCACAC/3’digoxigenin / |
