# Supplementary material for: Synergy between RecBCD subunits is essential for efficient DNA unwinding
Source: eLife. 2019 Jan 2;8:e40836. doi: 10.7554/eLife.40836 (PMC6338465; doi:10.7554/eLife.40836)
Supplement: Supplementary file 6. [file elife-40836-supp6.docx]

**Supplementary Table 6:** Results of fitting RecD force velocity curves to a Brownian ratchet before hydrolysis model, in complex and isolated.

| Parameter | Isolated RecD | RecD in complex |
| --- | --- | --- |
| $\Delta G_{RecD}\left( k_{b}T/bp \right)$ | $0.6\pm0.1$ | $-$ |
| $k_{b}\left( sec^{-1} \mu M^{-1} \right)$ | $5\pm1$ | $10\pm2$ |
| $k_{-b}\left( sec^{-1} \right)$ | $(1.2\pm0.3)\times{10}^{4}$ | $\left( 3\pm0.3 \right)\times{10}^{4}$ |
| $k_{c}^{eff}\left( sec^{-1} \right)$ | $335\pm12$ | $502\pm51$ |
| $K_{eq,tr}=\frac{k_{+tr,0}}{k_{-tr,0}}$ | $12\pm5$ | $2530\pm320$ |
| $\delta\left( bp \right)$ | $3.2\pm0.5$ | $3\pm0.3$ |
| $x^{\ddagger} (bp)$ | $-$ | $2.8\pm0.2$ |
